# Development and validation of motivators for medical specialist career choice questionnaire (MMSCCQ) - a methodological study

**DOI:** 10.1186/s12909-022-03523-3

**Published:** 2022-06-20

**Authors:** Anuradha Nadarajah, Shamala Ramasamy, Pathiyil Ravi Shankar, Chandrashekhar T. Sreeramareddy

**Affiliations:** 1 School of Postgraduate Studies, International Medical University, Kuala Lumpur, Malaysia; 2 Department of Psychology, International Medical University, Kuala Lumpur, Malaysia; 3 IMU Centre for Education, International Medical University, Kuala Lumpur, Malaysia; 4 Department of Community Medicine, International Medical University, No.126, Jalan Jalil Perkasa 19, Bukit Jalil, 57000 Kuala Lumpur, Malaysia

**Keywords:** Career choice, Choice of specialist training, House officers, Junior doctors, Medical specialties, Motivation

## Abstract

**Introduction:**

A validated instrument to assess the motivating factors influencing junior doctors’ medical specialist career choices is not available. The Motivators for Medical Specialist Career Choice Questionnaire (MMSCCQ) was developed and validated in the present study.

**Methods:**

An exploratory sequential mixed-methods study was conducted among house officers (HO) of a tertiary care hospital. A literature review was used to construct an interview guide. Seven HOs participated in an online, one-on-one audio-recorded in-depth interview (IDI). Seven sub-themes and 33 codes identified by thematic analyses were used to develop the MMSCCQ. The importance of each motivator was rated on a five-point Likert scale. The MMSCCQ was pretested, and a random sample of 262 house officers was invited to participate in an online survey. Psychometric evaluation was done using reliability statistics, and exploratory and confirmatory factor analyses.

**Results:**

The seven main themes identified by thematic analyses were labeled as factors related to ‘work schedule and personal life,’ ‘training opportunities’, ‘past work experiences’, ‘specialty characteristics’, ‘career prospects’, ‘patient care characteristics’, and ‘social factors.’ The highest ratings were given to “previous job experience” and “patient care traits. “The response rate was 71%, the mean age of the 185 HOs was 26.7 years (SD = 1.6). Females made up 63.8% of the population. The internal consistency for the overall questionnaire measured by Cronbach’s alpha was 0.85. Each construct demonstrated an acceptable internal consistency. Twenty-six of 33 items were maintained after an exploratory factor analysis was conducted, yielding 7 constructs with a 64.9% variance. Confirmatory factor analyses established the construct validity.

**Conclusion:**

The MMSCCQ has acceptable reliability and construct validity. Further studies are needed to test psychometric properties in different settings.

**Supplementary Information:**

The online version contains supplementary material available at 10.1186/s12909-022-03523-3.

## Background

The health workforce is a vital component of a health system. To meet the demand for healthcare services and the suggested doctor-to-population ratio requires a requisite number of medical professionals [1]. Young medical graduates such as house officers (HO) form the backbone of the future medical specialist health workforce. HO enter various medical specialties and subspecialties and exhibit interest in a variety of professional career paths for future medical practice [2]. However, their choices need not necessarily be aligned with the service requirements of their region or country or to the local disease burden [3, 4]*.*

Cross-sectional questionnaire survey studies among medical students and interns/house officers have reported that the choice of medical specialist career is influenced by several factors. These factors are the prestige of the specialty, income from the specialty, a preference for hospital-based practice in urban locations, experience during clinical postings, academic role models, career progression prospects, the flexibility of work arrangements, and work-life balance [5, 6]. Advice from family, friends, physicians, personal life events, and an interest in working in community-based settings may also influence their choice [7]^.^ Medical specialty choice and the factors influencing it differ by country and participants (medical students, interns, and house officers) [7–9]*.* A cross-sectional survey design using a questionnaire is suitable for identifying the choice of specialty career. However, limited items jjincluded in the questionnaire fail to provide a deeper exploration of the perspectives of medical students or junior doctors about motivations for their choice. Hence, it is critical to investigate the reason(s) as well as the decision-making process for medical specialty choices using qualitative studies.

Understanding the motivators that influence medical specialist career choices is critical for offering career advice and counselling to young medical graduates who are considering specializations that may not appear particularly enticing to them [10]. Qualitative studies have provided deeper insights into the factors that influence medical specialist career choices of medical students or graduates [11–13]. According to systematic reviews, most published studies employed non-validated ad hoc questionnaires to explore reasons or motivations for medical specialist career choices [5, 6]. Considering this, the Career Preference Factors Scale (CPFS) was developed to assess the motivators that drive the decision process of medical graduates about their specialist career choice [14]*.* However, this scale does not cover all the factors in detail, and only included the items suggested by previous questionnaire surveys and the process of scale development and validation was not reported. Thus, up till now a validated instrument to assess the motivators for medical specialist choice is unavailable. We used a robust research approach to develop an instrument that measures the motivators for medical specialist career choices. The process of developing and validating the Motivators for Medical Specialist Career Choice Questionnaire (MMSCCQ) is described in this paper.

## Methods

An instrument was developed and validated to assess the motivators for medical specialist career choice among medical graduates. This study was conducted in four phases.

**Phase 1**: A brief review of the literature to guide the qualitative interviews.

**Phase 2:** Qualitative study to identify main themes and sub-themes for generating items for the questionnaire.

**Phase 3:** Instrument development.

**Phase 4:** Psychometric evaluation.

### Phase 1

A search was carried out in PubMed, Scopus, Embase and Google Scholar databases to identify the relevant literature using the search terms ‘Medical Specialties’, ‘Career Choice’, and ‘Motivation’. Studies conducted among medical students and/or medical graduates about their intended medical specialist career choice and motivators for the choice of medical specialty were selected. The reported information about motivational factors and reasons for specialist choice were reviewed to be included as probes for the interview guide in the qualitative study. The items to be included as probes were discussed among the authors (AN and CTS) until a consensus was reached. Additional items to be included as probes were also derived from published systematic reviews and individual studies reported from Malaysia [5–7].

### Phase 2

A self-developed semi-structured topic guide was used for qualitative in-depth interviews (IDI). Online interviews using Zoom or Skype were preferred instead of face-to-face interviews due to the ongoing coronavirus disease (Covid-19) pandemic. Twelve house officers (HO) from Hospital Selayang, Selangor, Malaysia were selected for IDI and e-mail invitations sent. They were purposively selected to represent the three race groups (Malay, Chinese and Indian), and both genders. The interview questions focused on participants’ opinions about the existing specialty training opportunities in Malaysia, their interest in undertaking specialist career training in the future, preferred medical specialist choices and motivational factors, and reasons for the preferred choices of intended specialist training. Following the recommended IDI procedures each online interviews lasted for 25–35 minutes. IDIs were conducted until data saturation was achieved by the seventh participant when no new topics emerged. The recorded IDI were transcribed verbatim using Microsoft Office 365 dictation-transcription tool, but some manual corrections of transcripts were required for some recordings. Coding was done for each transcript and thematically analyzed with the aid of NVIVO software version 1.0 (2020). To ensure the rigor or trustworthiness, a summary of the analyses and transcripts of each interview was shared with the interviewees, and they were asked to review and provide affirmation or modifications, if any. The thematic analyses were done after anonymizing the transcripts. Initial reading and repeated readings of the transcript were done to obtain a general understanding, comprehension, and familiarization before coding. Constant comparison of themes and refinement were conducted along with the ongoing analyses. A rich and thick description was also used. Questionnaire items were developed using themes and sub-themes based on the responses to questions about the motivational factors and/or reasons for the intended medical specialist career choice.

### Phase 3

#### Instrument development

A pool of items was generated based on the thematic analysis. An initial draft questionnaire developed based on the qualitative study results consisted of a total of 33 items distributed under seven constructs (appendix A). The participants were asked to indicate the importance of each item (motivational factors/reasons) for their most preferred choice of intended medical specialty training on a 5-point Likert scale “not important at all” (1) to “very important” (5). The seven constructs and the number of items under each construct were ‘work schedule’ (A1–3); ‘patient care characteristics’ (B4–8); ‘specialty characteristics’ (C9–15); ‘personal reasons/factors’ (D16–21); ‘past work experience’ (E22–25); ‘training factors’ (F26–29); and ‘career prospects’ (G30–33).

### Phase 4

For psychometric evaluation, a cross-sectional online survey was conducted.

### Participants and methods

#### Pretest

A pre-test and comprehensibility check were conducted on a convenience sample of 30 house officers posted in hospitals other than the study site using WhatsApp messenger. Snowballing methods were used to recruit further participants. They were requested to check the comprehensibility of the questions with an open-ended section for feedback. They were also requested to consent to participate in a re-test 2 weeks later. All participants reported that the questions were clear and easily understood and no suggestions were made for any corrections. The same set of questions were distributed after a two-week interval. Only 12 respondents from the pre-test group completed the retest.

#### Survey data collection

A cross-sectional online survey was conducted among house officers posted in Hospital Selayang, Selangor, Malaysia during October and November 2020. An anonymized list of house officers and their electronic mails and mobile phone numbers registered with WhatsApp chat messenger was obtained from the human resource department. From the list of 364 HO with their contact details, 12 HO who had participated in the IDI were excluded. Every second house officer was selected using a systematic sampling from an ordered frame of every K^th^ participant. (total number of HO/minimum sample needed) N/n i.e., 352/200 ~ 2. As the frame was 2 every second participant was chosen. To obtain a random sample every second house officer was selected from the list. The questionnaire was designed using Google forms. A study information sheet and declaration of consent were sent to the selected participants via e-mail or WhatsApp messenger prior to the actual survey. The online questionnaire link was sent to those house officers who provided consent. To increase the response rate, a food voucher worth Ringgit Malaysian (RM) 5 (1 RM = 0.24 USD) was offered as an incentive. The survey link was available for 4 weeks, and non-responders received reminders on days 3 and 5, as well as weekly until the end of the four-week period. At the end of the fourth week, 185 of the 262 house officers had completed the online survey.

#### Data analyses

Raw data from Google forms were extracted into Microsoft Excel and imported into Statistical Package for Social Sciences (SPSS) version 25 for analysis. Descriptive statistics were computed. Data were grouped into seven constructs initially based on the seven sub-themes identified from IDI. The mean, standard deviation, range, skewness, and kurtosis of continuous data for each item and construct scores of the questionnaire were computed.

For reliability analysis**,** Cronbach’s alpha, inter-item correlation, corrected item-total correlation, and Cronbach’s alpha if item deleted were estimated. Corrected item total correlation shows a value of > 0.285. According to Kaplan & Saccuzzo (2013) a value > 0.30 is considered coherent between an item and the rest of the items in the questionnaire [15]. According to Hair, Babin. Anderson and Black (2018), an acceptable range of values for inter-item correlations is 0.30–0.90 [16]. Cronbach’s alpha measures internal consistency and a value greater than 0.7 indicates adequate internal consistency [17, 18]. To examine the test-retest reliability Pearson’s correlation coefficients and intraclass correlation coefficients (ICC) were estimated and a value greater than 0.75 indicated stability or acceptable test-retest reliability.

Exploratory factor analysis (EFA) was employed for content validation of the questionnaire. The main objective of using EFA is to retain the meaningful variables from the scale items extracted into the factor structure. Principal component analysis (PCA) extraction with varimax rotation was used in this analysis for 33 scale items with a sample size of 185 participants. The sample size was sufficient as according to Bryant et al. (1995), for every 1 item, a minimum of 5 responses are required [19, 20]. Kaiser-Meyer-Olkin (KMO) measure of sampling adequacy generated a middling threshold (0.73), indicating that the proportion of variance indicates the data is suitable for EFA [21]. Bartlett’s test of sphericity showed a significant *p*-value (χ^2^ = 1900.82, *p* < 0.001); both these findings ensured that the probability of correlation matrix having significant correlations was high and were a pre-requisite for performing an EFA. As the correlation between the factors was not anticipated, orthogonal rotation (namely varimax) was the optimal analysis model. The criterion set for inclusion of items in the model were 1) a limit for loading (> 0.5 retained); 2) cross loading was set at ≥0.5; 3) only stable factors with at least 3 items and Eigenvalue more than 1 were retained. The process was repeated until a stable structure was achieved.

A measurement model was analysed using Structural Equation Modelling (SEM). SEM is a confirmatory method providing the means for assessing and modifying the measurement model of latent constructs. The validity was evaluated through an assessment of model fit indices for construct validity. Several model fit indices were used to select the best model fit.

## Results

### Qualitative results

From the thematic analyses of audio recordings of the IDIs, the two major themes that emerged were HO “perceptions on specialist training opportunities in Malaysia” and “motivational factors for career specialty choices”. The seven sub-themes with the 33 refined codes for the major themes for motivators for intended medical specialist career choice were utilized as the questionnaire items for the instrument. A total of 33 scale items were generated for the Motivators for Medical Specialist Career Choice Questionnaire (MMSCCQ). Appendix B shows the item pool generation based on IDIs with the HO.

#### Quantitative results

##### Descriptive information

The response rate was 71% (185/262). A response rate of > 60% is considered adequate for a survey [22]*.* Mean age of the respondents was 26.7 years (SD, 1.6), and the range was 23–34 years. Females made up 63.8% and males 36.2% of respondents. About two-thirds of them were single, and 15.7% were married or in a relationship. Malay race made up 51.4% followed by Chinese (24.9%) and Indian (24.9%). More than half of them (58.9%) had graduated from a private medical school.

Mean scores for all the constructs were more than 3.0 and in the range of 3.4–4.2 (maximum score being five) (Table 1). The constructs having the highest and lowest mean scores were “past work experience” and “patient care characteristics” respectively. For individual items, the highest and lowest mean scores were “personal interest” (4.5, SD = 0.69) and “social media or public figure influence” (2.4, SD = 1.2) respectively. Out of the 33 items, 29 items yielded mean scores of ≥3, while the remaining 4 items had mean scores ranging from 2.4 to 2.9.

**Table 1:** Mean score for the motivational factors of career specialty preferences according to seven constructs (based on subthemes) and 33 scale items

Construct and items	Item Mean Score (SD)	Construct Mean Score (SD)
**A. Work schedule (items 1–3)**		**3.53 (0.865)**
A1. No on calls or less hectic	3.55 (1.122)	
A2. Shift work	3.26 (1.166)	
A3. Fixed working hours	3.78 (1.087)	
**B. Patient care characteristics (items 4–8)**		**3.38 (0.698)**
B4. Multidisciplinary or variety of illnesses/cases	3.70 (1.091)	
B5. Acute patient care	3.41 (1.231)	
B6. Minimal interactions with patient	2.56 (1.136)	
B7. Quick results/recovery after intervention or treatment	3.63 (1.096)	
B8. Continuous patient care	3.58 (1.121)	
**C. Specialty characteristics (items 9–15)**		**3.48 (0.652)**
C9. Challenging nature of the field	3.63 (1.008)	
C10. Medical based	3.49 (1.216)	
C11. Surgical based	2.88 (1.281)	
C12. Specialty with less medicolegal issues	3.62 (1.146)	
C13. Involves more hands-on skills and procedures	3.71 (1.188)	
C14. Flexible working conditions	3.98 (0.941)	
C15. Prestige or reputation of the specialty	3.07 (1.273)	
**D. Personal factors (items 16–21)**		**3.57 (0.602)**
D16. Family or relative influences/advice	2.60 (1.344)	
D17. Better work life balance	4.06 (1.028)	
D18. Personal interest in the specialty	4.52 (0.692)	
D19. Job satisfaction	4.44 (0.750)	
D20. Medical school experiences	3.45 (1.113)	
D21. Social media or public figure influence	2.35 (1.194)	
**E. Past work experience (items 22–25)**		**4.17 (0.707)**
E22. Good teamwork in the department	4.34 (0.786)	
E23. Events or defining moments during housemanship	3.84 (1.064)	
E24. Guidance and teaching activities in the department	4.20 (0.865)	
E25. Specialist or senior colleague role model/influences	4.31 (0.846)	
**F. Training factors (items 26–29)**		**3.92 (0.758)**
F26. Availability of parallel pathway	4.03 (0.935)	
F27. Availability of preparatory/training courses	4.17 (0.775)	
F28. Length of training (short)	3.71 (1.037)	
F29. Cost of training (less expensive)	3.75 (0.996)	
**G. Career prospects (items 30–33)**		**3.79 (0.771)**
G30. Future opportunities in private sector or private practice	4.01 (0.986)	
G31. Financially rewarding	3.73 (1.012)	
G32. Various subspecialties in the field to venture in future.	3.73 (1.075)	
G33. Future teaching opportunities	3.69 (1.037)	

#### Psychometric analysis

##### Reliability analysis

For the 33 scale items, the findings for most constructs revealed moderate to high reliability. The interpretation of the test-retest was restricted by the small number of respondents (Table 2). With regards to reliability, the Cronbach Alpha was 0.83, indicating the newly developed questionnaire had good internal consistency. Inter-item correlations were within an acceptable range except for a few items. Since CITC value of 0.285 for Item 1 is marginal, this value is negotiable. In addition, the Cronbach Alpha if Item Deleted value is 0.723, less than the overall reliability, hence it was decided to retain this item.

**Table 2:** Reliability statistics, Cronbach’s alpha and test-retest reliability

Construct (Number of Items)	Cronbach’s alpha (0.85)	Range of inter-item correlation	Corrected Item-Total Correlation	Cronbach’s Alpha if Item Deleted	Test-retest reliability (*n* = 12)			
					*r*	*p*-value	ICC	*p*-value
Work schedule (3)	0.655	0.238–0.511	0.285	0.723	0.960	0.000	0.978	< 0.001
Patient care (5)	0.587	- 0.021 – 0.506	0.388	0.689	0.314	0.320	0.476	0.150
Specialty characteristics (7)	0.643	- 0.271 - 0.576	0.491	0.666	0.292	0.208	0.563	0.093
Personal factors (6)	0.596	- 0. 025–0.717	0.554	0.655	0.498	0.099	0.637	0.054
Past work experiences (4)	0.797	0.329–0.708	0.443	0.676	0.212	0.508	0.299	0.283
Training factors (4)	0.820	0.443–0.669	0.397	0.688	0.334	0.288	0.477	0.148
Career prospects (4)	0.741	0.209–0.666	0.475	0.667	0.728	0.007	0.836	0.003

### Exploratory factor analysis

EFA resulted in the extraction of seven factors from the scree plot and these explained cumulative total variance of 64.9% with eigenvalues between 1.27 and 5.42. Item number B6 was removed due to the factor loading value being < 0.5, indicating a less well-defined structure. Items numbered A2, B7, C10, D18, D19 and G33 were removed as the initial structure formed an unstable factor with < 3 items in one component. In the final structure seven factors were extracted with 26 items presenting a total variance of 64.9%. Factor loading for all these items was above 0.50, which is considered as a well-defined structure. Most of the retained items of each construct loaded consistently with the initial construct from the qualitative findings except for some items in “personal factors” construct (items D16, D17, D21) and “specialty characteristics” construct (items: C12, C14, C15) that were loaded in other constructs instead of on their initial construct. Eventually, a total of 26 items were retained and 7 items were removed. The factors were relabeled accordingly. Table 3 shows the summary results of the exploratory factor analyses.

**Table 3:** Summary of exploratory factor analysis of motivational factors for career specialty preferences

Construct	Retained items	Factor loading	Variance (%)	Mean (SD)	Eigen value
**Construct 1** Work Schedule and personal factors	A1. No or less hectic on calls	0.755	20.8	3.6 (0.75)	5.42
	A3. Fixed working hours	0.716			
	C12. specialty with less medicolegal risk	0.584			
	C14. Flexible working conditions	0.613			
	D16. Family or relative influences/advice	0.548			
	D17. Better work life balance	0.725			
**Construct 2** Patient care characteristics	B4. Multidisciplinary or variety of illnesses/cases	0.796	5.5	0.82 (0.85)	1.43
	B5. Acute patient care	0.644			
	B8. Continuous patient care	0.740			
**Construct 3** Training Factors	F26. Availability of parallel pathway	0.798	11.2	3.92 (0.76)	2.91
	F27. Availability of training courses	0.721			
	F28. Length of training (short)	0.798			
	F29. Cost of training (less expensive)	0.773			
**Construct 4** Past work experience	E22. Good teamwork in the department	0.593	10.0	4.13 (0.74)	2.60
	E23. Events or defining moments during housemanship	0.701			
	E24 Guidance and teaching activities in the department	0.796			
	E25.Specialist or senior colleague role model/influences	0.866			
**Construct 5** Specialty characteristic	C 9. Challenging nature of the field	0.552	6.4	3.56 (0.91)	1.65
	C11. Surgical based	0.710			
	C13.More hands-on skills and procedures	0.888			
**Construct 6** Career prospects	G30. Future opportunities in private sector or private practice	0.787	6.1	0.67 (0.81)	1.59
	G31. Financially rewarding	0.798			
	G32. Various subspecialties in the field to venture in future	0.704			
**Construct 7** Social factors	C15. Prestige or reputation of the specialty	0.654	4.9	2.96 (0.93)	1.21
	D20. Medical school experiences	0.746			
	D21. Social media or public figure influence	0.753			
Total variance: **64.9%** Overall Cronbach’s alpha **0.830**					

Seven (7) items were removed:

Factor loading < 0.5

B6 Minimal interaction with patient (factor loading = 0.385)

Items that form unstable constructs (less than 3 items in one factor)

B7: Quick results/recovery after intervention

C10: Medical based

D18. Personal interest in the specialty

D19: Job satisfaction

A2: Shift work

G33: Future teaching opportunities

### Confirmatory factor analysis

The Chi Square Test of Goodness of Fit yielded a significant value; χ^2^ **=** 0.624 (*df* = 278), < 0.001, which was below the threshold of 0.05 (reported if *N* > 200). Additionally, the value of Goodness of Fit Index = 0.957, Comparative Fit Index (CFI) = 0.957, Tucker-Lewis Index (TLI) = 0.950, Non-normed Fit Index (NNFI) = 0.950, Parsimony Normed Fit Index (PNFI) 0.791, and Incremental Fit Index (IFI) = 0.957 showed moderate fit of the model. The factor loadings are displayed in Table 4. All the factor loadings yielded a value of > 0.50, indicating no items needed to be removed. The highest factor loading was for Item E34 = 0.92 whereas the lowest was for A1 and D20 = 0.51. The results infer that construct validity was well established for this questionnaire.

**Table 4:** Items, factor loading and measurement error from confirmatory factor analyses

Constructs	Items	Factor Loading	Measurement Error
**Construct 1** Work schedules and personal factors	A1. No or less hectic on calls	0.51	0.74
	A3. Fixed working hours	0.56	0.69
	C12. specialty with less medicolegal risk	0.61	0.63
	C14. Flexible working conditions	0.81	0.35
	D16. Family or relative influences/advice	0.56	0.69
	D17. Better work life balance	0.75	0.43
**Construct 2** Patient care characteristics	B4. Multidisciplinary or variety of illnesses/cases	0.71	0.49
	B5. Acute patient care	0.81	0.34
	B8. Continuous patient care	0.61	0.63
**Construct 3** Training factors	F26. Availability of parallel pathway	0.82	0.32
	F27. Availability of training courses	0.88	0.22
	F28. Length of training (short)	0.80	0.36
	F29. Cost of training (less expensive)	0.74	0.45
**Construct 4** Past work experience	E22. Good teamwork in the department	0.69	0.52
	E23. Events or defining moments during housemanship	0.73	0.47
	E24 Guidance and teaching activities in the department	0.92	0.15
	E25.Specialist or senior colleague role model/influences	0.83	0.31
**Construct 5** Specialty characteristics	C 9. Challenging nature of the field	0.69	0.52
	C11. Surgical based	0.65	0.58
	C13.More hands-on skills and procedures	0.86	0.26
**Construct 6** Career prospects	G30. Future opportunities in private sector or private practice	0.85	0.28
	G31. Financially rewarding	0.84	0.29
	G32. Various subspecialties in the field to venture in future	0.69	0.54
**Construct 7** Social factors	C15. Prestige or reputation of the specialty	0.89	0.21
	D20. Medical school experiences	0.51	0.74
	D21.social media or public figure influence	0.63	0.60

## Discussion

Using a robust psychometric analysis, we developed and validated a new instrument MMSCCQ that comprehensively captures the motivators of HO for intended medical specialist career choice. MMSCCQ was designed using information about motivators across various main themes and sub-themes identified by a literature review and IDI. Overall, the content and construct validity of the instrument was acceptable as shown by exploratory followed by confirmatory factor analyses suggesting that MMSCCQ was psychometrically sound with an acceptable Cronbach’s alpha and range of inter-item correlations. Despite the variability in the number of items across the seven constructs all of them had good internal consistency and reasonably good test-retest reliability over a two-weeks interval. The robust process employed for item development, and content expert review ensured completeness and clarity of the items in the MMSCCQ.

During recent decades, there is a growing interest on training doctors in medical specialties for health workforce planning to match the demand for specialist services in the health system. The number and type of specialists required is also based on the existing number of specialists in each discipline and their spatial distribution [2, 4]. This has resulted in an ever-growing body of literature on medical specialist career choices among medical students and medical graduates [5, 6]. Yet, to date, to the best of our knowledge only two other studies have reported psychometric properties of questionnaires used to study the motivators for selected medical specialist career choices [14]. The Career Preference Factors Scale (CPFS) from Malaysia was developed mainly based on two studies from Canada applicable to career pathways available in Canada [23, 24]. MMSCCQ showed acceptable psychometric properties, 19 of the 33 items fell under five broad constructs of thematic network factors proposed based on literature review [5]. All the items generated from IDIs match those reported in the existing literature [5, 6, 10]. However, on EFA seven items were removed either due to ill-defined construct or instability, and some items from constructs of “personal factors” and “specialty characteristics” straddling to other constructs. A few of the items under these constructs also had lower inter-item correlations and were excluded after EFA. Retention of 26 items from the original 33 items and re-alignment of factors under different constructs reflect overlapping and duplicated main themes and sub-themes identified by ‘rich data’ and ‘thick description’ used for thematic analyses of IDIs. Nevertheless, the retained items are broadly reflective of existing motivators identified in the literature [5, 6, 10].

The constructs “work schedule and personal factors” had the highest factor loading while it was lowest for “social factors” such as “social media influence”, and “prestige of the specialty”. Among the ad hoc questionnaires developed in previous studies about the motivators for choice of specialty, only a few studies reported factor analyses to validate the motivators that determine the choice of medical specialties [25, 26]. In a study among Japanese medical students and junior doctors, “fulfilling life with job security”, “bioscientific orientation”, “personal reasons”, “advice from others”, “educational experience” were mentioned, and these varied across the choice of specialties. In a study among Swiss postgraduate doctors, “work and time-related aspects”, “career-related aspects” and “patient orientation” were the most important among the 19 factors listed [26]. Main motivators for medical specialist choice vary according to country since the available career pathways and training pathways are different in each country. Thus, the motivators for medical specialist career choice are context specific. Adapting the motivators based on previous literature often from a different context is not recommended. Our robust methodology provides a very wide range of constructs and items suitable to adapt in similar settings.

Some limitations inherent to the study sample and study participants should be considered while interpreting the development of this new instrument. Though the sample studied was adequate, our results need to be confirmed on a larger sample of HO. In particular, the sample who completed test-retest was very small and this may affect the stability of ICC and thus the inferences that can be drawn. A study from a single tertiary care hospital located in an urban locality would be more reflective of personal and professional motivators appropriate to an urban setting. Motivators and their choice are known to be different among students or house officers based on impressions created during clinical exposure in different medical specialties and are likely to change. Nevertheless, we identified broad constructs and items under each that can inform future survey research to comprehensively explore these motivators driving medical specialist career choice.

## Conclusion

In-depth interviews provided deeper insights into the several broad constructs of motivators for medical specialist career choice. The main themes and subthemes identified provided inputs for constructs and items in MMSCCQ. The MMSCCQ has adequate reliability, construct, and content validity. Further studies are needed to assess the psychometric properties of MMSCCQ before possible adaptation in comparable contexts and settings in future research.

## Supplementary Information

**Additional file 1.**

**Additional file 2.**

## Data Availability

The data sets generated during and/or analysed during the current study are available from the corresponding author on reasonable request.

## Abbreviations

MMSCCQ: Motivators for Medical Specialist Career Choice Questionnaire
CPFS: Career Preference Factors Scale
HO: House officers
IDI: In-depth interviews
RM: Ringgit Malaysian
SPSS: Statistical Package for Social Sciences
ICC: Intraclass Correlation Coefficients
EFA: Exploratory Factor Analysis
PCA: Principal Component Analysis
KMO: Kaiser-Meyer-Olkin
