# Supplementary material for: Development and validation of motivators for medical specialist career choice questionnaire (MMSCCQ) - a methodological study
Source: BMC Med Educ. 2022 Jun 20;22:474. doi: 10.1186/s12909-022-03523-3 (PMC9206890; doi:10.1186/s12909-022-03523-3)
Supplement: Supplementary file 1 — Additional file 1. [file 12909_2022_3523_MOESM1_ESM.docx]

**Motivating factors for career specialty preference:** This section is based on which reasons or factors motivate you to choose your most preferred specialty (1st choice) to specialize in the future.

Indicate to which extent the following factors are important for you in choosing your preferred choice of specialty.

For each item, please select the appropriate answer according to the importance scale/score between 1 to 5 as indicated below:

1: Not important at all

2: Not important

3: Somewhat important

4: Important

5: Very important

| **Motivational Factors / Reasons for the preferred choice of specialty** | | | | | | |
| --- | --- | --- | --- | --- | --- | --- |
| **A WORK SCHEDULE** | | **1** | **2** | **3** | **4** | **5** |
| 1 | No on calls or less hectic on calls |  |  |  |  |  |
| 2 | Shift work |  |  |  |  |  |
| 3 | Fixed working hours |  |  |  |  |  |
| **B PATIENT CARE CHARACTERISTICS** | | **1** | **2** | **3** | **4** | **5** |
| 4 | Multidiscipline or wide variety of illness/cases |  |  |  |  |  |
| 5 | Acute management care of patient |  |  |  |  |  |
| 6 | Minimal interaction with patient |  |  |  |  |  |
| 7 | Quick results/recovery after intervention or treatment is common in this field |  |  |  |  |  |
| 8 | Continuous patient care |  |  |  |  |  |
| **C SPECIALTY CHARACTERISTICS** | | **1** | **2** | **3** | **4** | **5** |
| 9 | Challenging nature of the field |  |  |  |  |  |
| 10 | Medical based |  |  |  |  |  |
| 11 | Surgical based |  |  |  |  |  |
| 12 | Less medicolegal issues |  |  |  |  |  |
| 13. | Involves more hands-on skill and procedures |  |  |  |  |  |
| 14. | Flexible working conditions |  |  |  |  |  |
| 15. | Prestige or reputation of the specialty |  |  |  |  |  |
| **D PERSONAL REASON /FACTOR** | | **1** | **2** | **3** | **4** | **5** |
| 16 | Family or relative influences/advice |  |  |  |  |  |
| 17 | Better work life balance |  |  |  |  |  |
| 18 | Personal interest |  |  |  |  |  |
| 19 | Job satisfaction |  |  |  |  |  |
| 20 | Medical school experiences |  |  |  |  |  |
| 21 | Social media or public figure influence |  |  |  |  |  |
| **E**  **PAST WORK EXPERIENCE** | | **1** | **2** | **3** | **4** | **5** |
| 22 | Good teamwork in the department |  |  |  |  |  |
| 23 | Critical events or defining moment during HO rotations related to the specialty |  |  |  |  |  |
| 24 | Guidance and teaching activities in the department |  |  |  |  |  |
| 25 | Specialist or senior colleague role model/ influences |  |  |  |  |  |
| **F TRAINING FACTORS** | | **1** | **2** | **3** | **4** | **5** |
| 26 | Availability of parallel pathway |  |  |  |  |  |
| 27 | Availability of preparatory /training courses either locally or distance learning/ online mode |  |  |  |  |  |
| 28 | Length of training (shorter training period) |  |  |  |  |  |
| 29 | Cost of training (less expensive) |  |  |  |  |  |
| **G CAREER PROSPECTS** | |  |  |  |  |  |
| 30 | Future opportunities in private sector or private practice |  |  |  |  |  |
| 31 | Financially rewarding |  |  |  |  |  |
| 32 | Variety of subspecialties in the field to venture |  |  |  |  |  |
| 33 | Future teaching opportunities |  |  |  |  |  |
