# Supplementary material for: Development and validation of motivators for medical specialist career choice questionnaire (MMSCCQ) - a methodological study
Source: BMC Med Educ. 2022 Jun 20;22:474. doi: 10.1186/s12909-022-03523-3 (PMC9206890; doi:10.1186/s12909-022-03523-3)
Supplement: Supplementary file 2 — Additional file 2. [file 12909_2022_3523_MOESM2_ESM.docx]

**Raw data on item pool generation for the “motivational factors of career specialty preference’ scale items in questionnaire:**

| **THEME 2 : MOTIVATIONAL FACTORS FOR CAREER SPECIALTY PREFERENCE** | | | | | |
| --- | --- | --- | --- | --- | --- |
| Open Coding/Free codes identified from transcripts  (managed in NVivo ) | Refined codes with grouping | Refinement of codes with revised grouping under main themes | Initial subthemes | **33 ITEMS GENERATED FROM REFINED CODES AND QUOTATION**  **(33 scale items)** | **REVISED TERMINOLOGY FOR SUBTHEMES / QUESTIONNAIRE DOMAIN**  **(7domains)** |
| - no on calls - flexible post call off - reasonable on calls + Less hectic on calls - Shift work - Fixed working hours - multidiscipline or variety of illness/cases handled - Short term management and no long term commitment with patient? Rephrase to acute - Frontline nature of work – rephrased acute - Minimal interaction with patient - Continuous patient care - Challenging nature of the field - Quick results after intervention or treatment - Job content- comprised in specialty characteristics - medical based - surgical based - Less medicolegal issues - Involve hands on skill or experience - No ward rounds -omitted-cause similar like minimal interaction with patient? - Flexible working - Prestige - Family or relative influences/advice - Better work life balance - Personal interest - Interest in Urgent care- omit/ changed (refined acute care) - Job satisfaction - Medical school experiences - Social media or public figure influence - Good teamwork in the department - Critical events and defining moment - Guidance and teaching activities in the department - Working with skillful staffs in the department -combine as teamwork - Work like family – combine with team work - Specialist approachable and teach - specialist influence /guidance and teaching activities in department - Specialist or senior colleague’s influences/role model - HO posting experiences – renamed events/defining moment during HO - Personal or pleasant working experience🡪 - availability of recognized alternate / parallel pathway - Availability of preparatory programs locally - Availability of distance learning/ online training programs - Length of training(shorter training period) - Less expensive cost of training - Future opportunities in private sector - Private practice – combine with private sector - Financial reward - Variety of subspecialties in the - Can conduct courses – teaching opportunities | no on calls  flexible post call off  reasonable on calls  Less hectic on calls  (On call) | - No or less hectic on calls - Shift work - Fixed working hours   (timing of work) | Timing of work | **A1 No or less hectic on calls**  **A2 Shift work**  **A3 Fixed working hours** | **A WORK SCHEDULE** |
|  | Shift work  Fixed working hours  ( working hour) |  |  |  |  |
|  | - multidiscipline or wide variety of illness/cases handled - Acute management of patient - Minimal interaction with patient - Continuous patient care   (nature of patient care) | - multidiscipline or wide variety of illness/cases handled - Acute management of patient - Minimal interaction with patient - Continuous patient care - Quick results/recovery after intervention or treatment common after recovery   (nature of patient care) | Nature of patient care | **B4 Multidiscipline or wide variety of cases handled**  **B5 Acute patient care**  **B6 Minimal interaction with patient**  **B7 Continuous patient care**  **B8 Quick results/recovery after intervention** | **B PATIENT CARE CHARACTERISTICS** |
|  | - Challenging nature of the field - medical based - surgical based - Less medicolegal issues - Involve hands on skill or experience - Flexible working conditions - Prestige/reputation of the specialty - **Quick results/recovery after intervention or treatment (shift to theme 2)**   **(job content/ characteristics)** | - Challenging nature of the field - medical based - surgical based - Less medicolegal issues - Involve hands on skill or experience - Flexible working conditions - Prestige/reputation of the specialty   (Specialty characteristics) | Specialty characteristics | **C9 Challenging nature of the field**  **C10 Medical based**  **C11 Surgical based**  **C12 Less medicolegal issues**  **C13 Involves more hands-on skill or experience**  **C14 Flexible working conditions**  **C15Prestige/reputation of the specialty** | **C SPECIALTY**  **CHARACTERISTICS** |
|  | - Family or relative influences/advice - Better work life balance - Personal interest - Job satisfaction - Medical school experiences - Social media or public figure influence   ( Personal factors/ reasons) | - Family or relative influences/advice - Better work life balance - Personal interest - Job satisfaction - Medical school experiences - Social media or public figure influence     (Personal factors/ reasons) | Personal factors/ reasons | **D16 Family or relative influences/advice**  **D17 Better work life balance**  **D18 Personal interest**  **D19 Job satisfaction**  **D20 Medical school experiences**  **D21 Social media or public figure influence** | **D PERSONAL FACTORS** |
|  | - Good teamwork in the department - Events/ defining moment during HO rotations - Guidance and teaching activities in the department - Specialist or senior colleagues influences/role model   **(Working experiences in the department)** | - Good teamwork in the department - Events/ defining moment during HO rotations - Guidance and teaching activities in the department - Specialist or senior colleagues influences/role model   **(Working experiences/condition in the department)** | (Working experiences/condition in the department) | **E22 Good teamwork in the department**  **E23 HO posting experiences**  **E24 Guidance and teaching activities in the department**  **E25 Specialist or senior colleagues influences/role model** | **E PAST WORKING EXPERIENCE** |
|  | - availability of recognized alternate / parallel pathway - **Availability of preparatory/training programs locally** - **Availability of distance learning/ online training/preparatory courses 🡪 combine with above.** - Length of training (shorter training period) - cost of training (less expensive)   **(specialist training courses/education factors)** | - availability of recognized alternate / parallel pathway - Availability of preparatory/training programs locally - Availability of distance learning/ online training/preparatory courses 🡪 combine with above.   🡪Length of training (shorter training period)  🡪cost of training (less expensive)  (specialist training courses/education factors) | specialist training courses/education factors | **F26 Availability of parallel pathway**  **F27 Availability of preparatory/training**  **Courses**  **F28Length of training (shorter training period)**  **F29 cost of training (less expensive)** | **F TRAINING FACTORS** |
|  | - Future opportunities in private sector or practice - Financially rewarding - Various subspecialties in the field to venture - Future teaching opportunities   (Prospect of career in the specialty) | - Future opportunities in private sector or practice - Financially rewarding - Various subspecialties in the field to venture - Future teaching opportunities   (Prospect of career in the specialty) | - **Prospect of career in the specialty)** | **G30 Future opportunities in private sector or practice**  **G31 Financially rewarding**  **G32 Various subspecialties to venture**  **G33 Future teaching opportunities** | **G CAREER PROSPECTS** |
